# Supplementary material for: Molecular detection of Orientia tsutsugamushi infection in bats from the China-Myanmar border
Source: PLoS Negl Trop Dis. 2026 Jan 12;20(1):e0013860. doi: 10.1371/journal.pntd.0013860 (PMC12818740; doi:10.1371/journal.pntd.0013860)
Supplement: S2 Table — (DOCX) [file pntd.0013860.s002.docx]

| **S2 Table.** The primers for the detection of Ot in this study | | | | |
| --- | --- | --- | --- | --- |
| Primer Name | Sequence (5'-3') | Tm(℃) | Amplification conditions | References |
| ST56KF1 | TACATTAGCTGCGGGTATGACA | 46 | **Detection of the 56-kDa TSA gene**: **First round**: Amplification with primers ST56KF1 and ST56KR1 (expected fragment size: 306-339 bp). The PCR conditions were as follows: pre-denaturation at 94°C for 3 min, followed by 30 cycles of denaturation at 94°C for 30 s, annealing at 51°C for 30 s, and extension at 72°C for 30 s, with a final extension at 72°C for 10 min and cooling to 10°C for 1 min. **Second round**: Nested amplification with primers ST56KF2 and ST56KR2 (expected fragment size: 150-168 bp). The cycling conditions were identical to the first round, except for an annealing temperature of 52°C and an extension time of 20 s. | [1] |
| ST56KR1 | CCAGCATAATTCTTCAACCAAG | 46 |  |  |
| ST56KF2 | GAGCAGAGCTAGGTGTTATGTA | 46 |  |  |
| ST56KR2 | TAGGCATTATAGTAGGCTGAGG | 47 |  |  |
| tsu-34 | TCAAGCTTATTGCTAGTGCAATGTCTGC | 58 | **Amplification of the 56-kDa TSA gene**: **First round**: Amplification with primers tsu-34 and tsu-55 (expected fragment size: 1013 bp). The PCR conditions were as follows: pre-denaturation at 94°C for 3 min, followed by 30 cycles of denaturation at 94°C for 30 s, annealing at 50°C for 30 s, and extension at 72°C for 1 min 10 s, with a final extension at 72°C for 10 min and cooling to 10°C for 1 min. **Second round**: Amplification with primers tsu-34 and tsu-11 (expected fragment size: 868 bp). The cycling conditions were identical to the first round, except for an annealing temperature of 48.3°C and an extension time of 30 s. | [2] |
| tsu-55 | AGGGATCCCTGCTGCTGTGCTTGCTGCG | 67 |  |  |
| tsu-11 | CTAGGGATCCCGACAGATGCACTATTAGGC | 64 |  |  |
| Otr47-263F | GTGCTAAGAAARGATGATACTTC | 51 | **Detection of the 47-kDa *htrA* gene**: **First round**: Amplification with primers Otr47-263F and Otr47-1133R (expected fragment size: 870 bp). PCR conditions: pre-denaturation at 94°C for 3 min, followed by 29 cycles of denaturation at 94°C for 30 s, annealing at 51°C for 30 s, and extension at 72°C for 1 min, with a final extension at 72°C for 10 min and cooling to 10°C for 1 min. **Second round**: Amplification with primers Otr47F and Otr47-1133R (expected fragment size: 786 bp). The cycling conditions were identical to the first round, except for an extension time of 50 s. | [3, 4] |
| Otr47F | TAAAGGTTAAGTTTATGAAAAAGGCATTT | 51 |  |  |
| Otr47-1133R | ACATTTAACATACCACGACGAAT | 51 |  |  |
| GroEL-14F | TTGTACATRGCGATCAATGTCGT | 53 | **Detection of the GroEL gene**: **First round**: Amplification with primers GroEL-14F and GroEL-1667R (expected fragment size: 1654 bp). PCR conditions: pre-denaturation at 94°C for 3 min, followed by 29 cycles of denaturation at 94°C for 30 s, annealing at 55°C for 30 s, and extension at 72°C for 2 min, with a final extension at 72°C for 10 min and cooling to 10°C for 1 min. **Second round**: Amplification with primers GroEL-14F and GroEL-772R (expected fragment size: 758 bp). The cycling conditions were identical to the first round, except for an extension time of 1 min. | [5] |
| GroEL-1667R | TAGAAATCCATTCCGCCCATAC | 55 |  |  |
| GroEL-772R | GAGCTTCTCCGTCTACATCATCAG | 55 |  |  |
| 16sO79F | ATTAATGCTGAGCTTGCTTAGCAT | 51 | **Detection of the 16S rRNA gene**: **First round**: Amplification with primers 16sO79F and 16sOR1198R (expected fragment size: 1120 bp). PCR conditions: pre-denaturation at 94°C for 3 min, followed by 29 cycles of denaturation at 94°C for 30 s, annealing at 51°C for 30 s, and extension at 72°C for 1 min 20 s, with a final extension at 72°C for 10 min and cooling to 10°C for 1 min. **Second round**: Amplification with primers 16s155F and 16sOR1198R (expected fragment size: 1043 bp). The cycling conditions were identical to the first round, except for an extension time of 1 min 10 s. | [6] |
| 16s155F | TCAGTACGGAATAACWTTTAGAAATAA | 51 |  |  |
| 16sOR1198R | TTTCCTATAGTTCCCGGCATT | 51 |  |  |
| Otsu47-kDa htrA FP630 | AACTGATTTTATTCAAACTAATGCTGCT | 51 | **Quantitative PCR (qPCR) targeting the 47-kDa htrA gene**: The amplification conditions comprised 50 cycles with pre-denaturation at 95°C for 5 min, followed by denaturation at 95°C for 10 s, annealing at 60°C for 30 s, and fluorescence signal acquisition times. | [7] |
| Otsu47-kDa htrA RP747 | TATGCCTGAGTAAGATACRTGAATRGAATT | 51 |  |  |
| Otsu47-kDa htrA Probe | FAM-TGGGTAGCTTTGGTGGACCGATGTTTAATCT-BHQ1 | 60 |  |  |

**References**

**1.** Zhang S, Song H, Liu Y, Li Q, Wang Y, Wu J, et al. Scrub typhus in previously unrecognized areas of endemicity in China. J Clin Microbiol. 2010;48(4):1241-4. <https://dx.doi.org/10.1128/JCM.01784-09> PMID:20129967

**2.** Furuya Y, Yoshida Y, Katayama T, Yamamoto S, Kawamura A, Jr. Serotype-specific amplification of *Rickettsia tsutsugamushi* DNA by nested polymerase chain reaction. J Clin Microbiol. 1993;31(6):1637-40. <https://dx.doi.org/10.1128/jcm.31.6.1637-1640.1993> PMID:8315007

**3.** Jiang J, Paris DH, Blacksell SD, Aukkanit N, Newton PN, Phetsouvanh R, et al. Diversity of the 47-kD HtrA nucleic acid and translated amino acid sequences from 17 recent human isolates of *Orientia*. Vector Borne Zoonotic Dis. 2013;13(6):367-75. <https://dx.doi.org/10.1089/vbz.2012.1112> PMID:23590326

**4.** Weitzel T, Aylwin M, Martinez-Valdebenito C, Jiang J, Munita JM, Thompson L, et al. Imported scrub typhus: first case in South America and review of the literature. Trop Dis Travel Med Vaccines. 2018;4:10. <https://dx.doi.org/10.1186/s40794-018-0070-8> PMID:30140442

**5.** Arai S, Tabara K, Yamamoto N, Fujita H, Itagaki A, Kon M, et al. Molecular Phylogenetic Analysis of *Orientia tsutsugamushi* Based on the *groES* and *groEL* Genes. Vector-Borne and Zoonotic Diseases. 2013;13(11):825-9. <https://dx.doi.org/10.1089/vbz.2012.1155> PMID:24107204

**6.** Abarca K, Martinez-Valdebenito C, Angulo J, Jiang J, Farris CM, Richards AL, et al. Molecular Description of a Novel Orientia Species Causing Scrub Typhus in Chile. Emerg Infect Dis. 2020;26(9):2148-56. <https://dx.doi.org/10.3201/eid2609.200918> PMID:32818400

**7.** Jiang J, Chan TC, Temenak JJ, Dasch GA, Ching WM, Richards AL. Development of a quantitative real-time polymerase chain reaction assay specific for *Orientia tsutsugamushi*. Am J Trop Med Hyg. 2004;70(4):351-6. <https://dx.doi.org/10.4269/ajtmh.2004.70.351> PMID:15100446
