# Supplementary figures and images for: Molecular detection of Orientia tsutsugamushi infection in bats from the China-Myanmar border
Source: PLoS Negl Trop Dis. 2026 Jan 12;20(1):e0013860. doi: 10.1371/journal.pntd.0013860 (PMC12818740; doi:10.1371/journal.pntd.0013860)

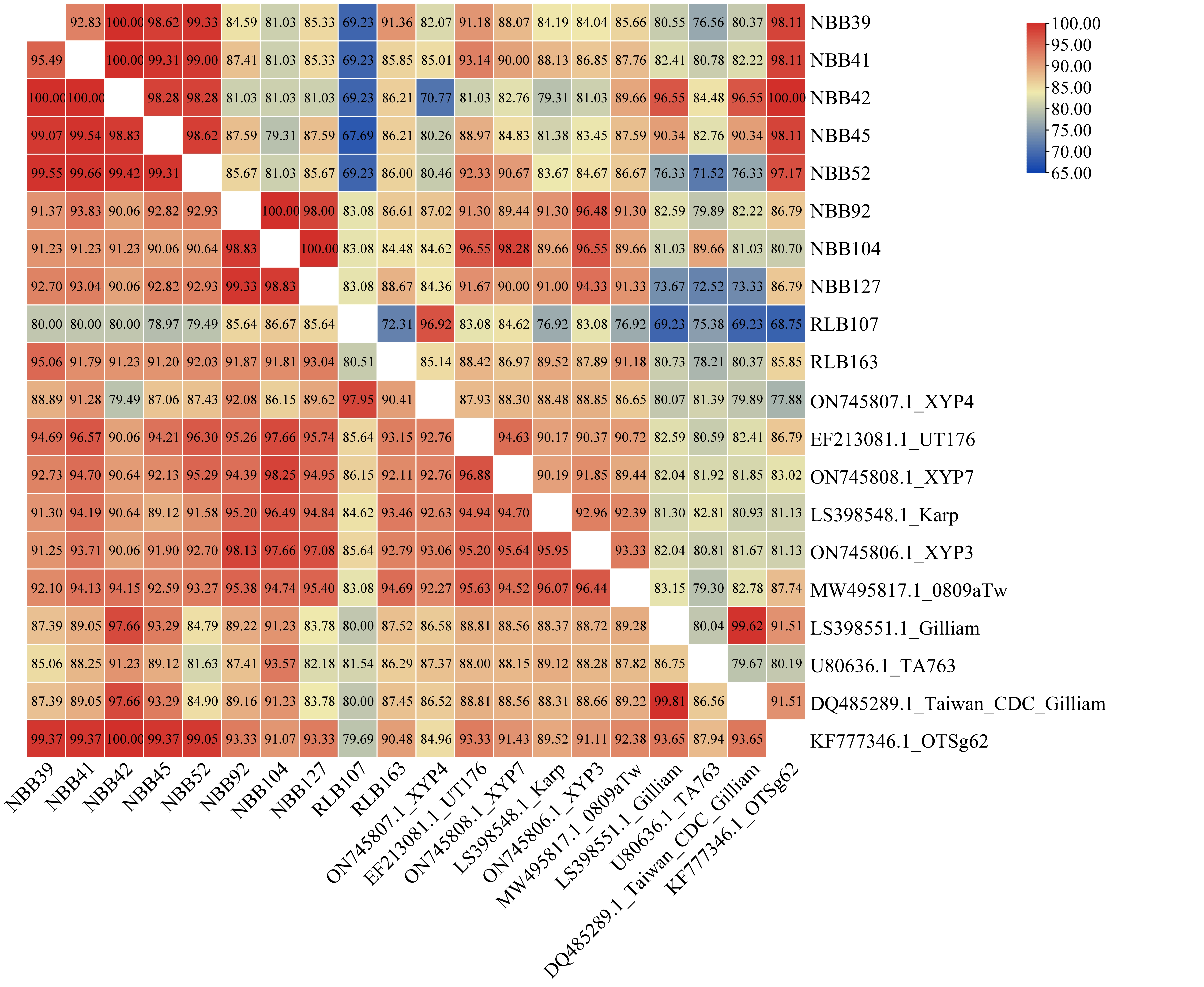

Supplement: S1 Fig — Top right indicates acid sequence identity, while bottom left shows nucleotide sequence identity. (TIF) [file pntd.0013860.s004.tif]

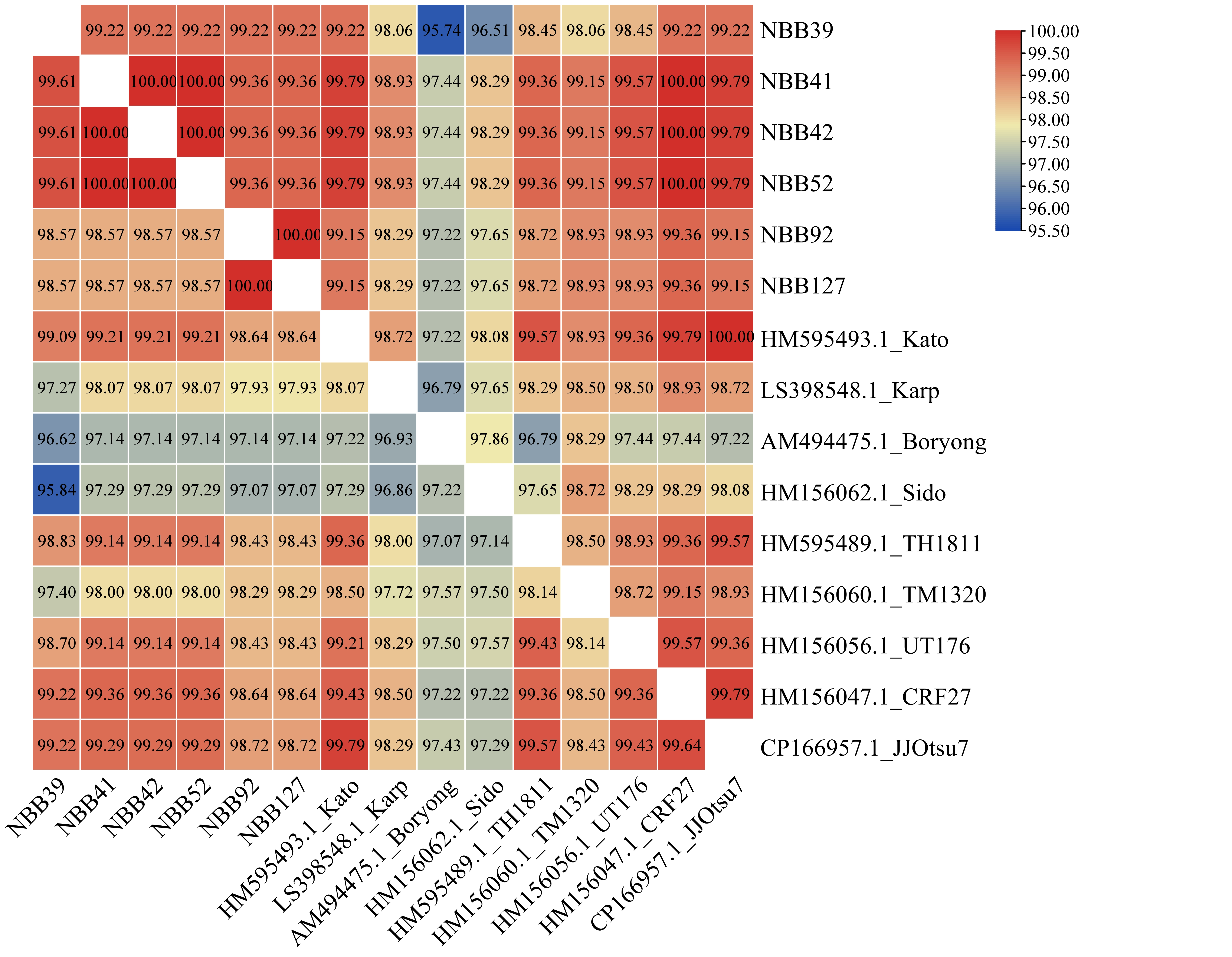

Supplement: S2 Fig — Top right indicates acid sequence identity, while bottom left shows nucleotide sequence identity. (TIF) [file pntd.0013860.s005.tif]

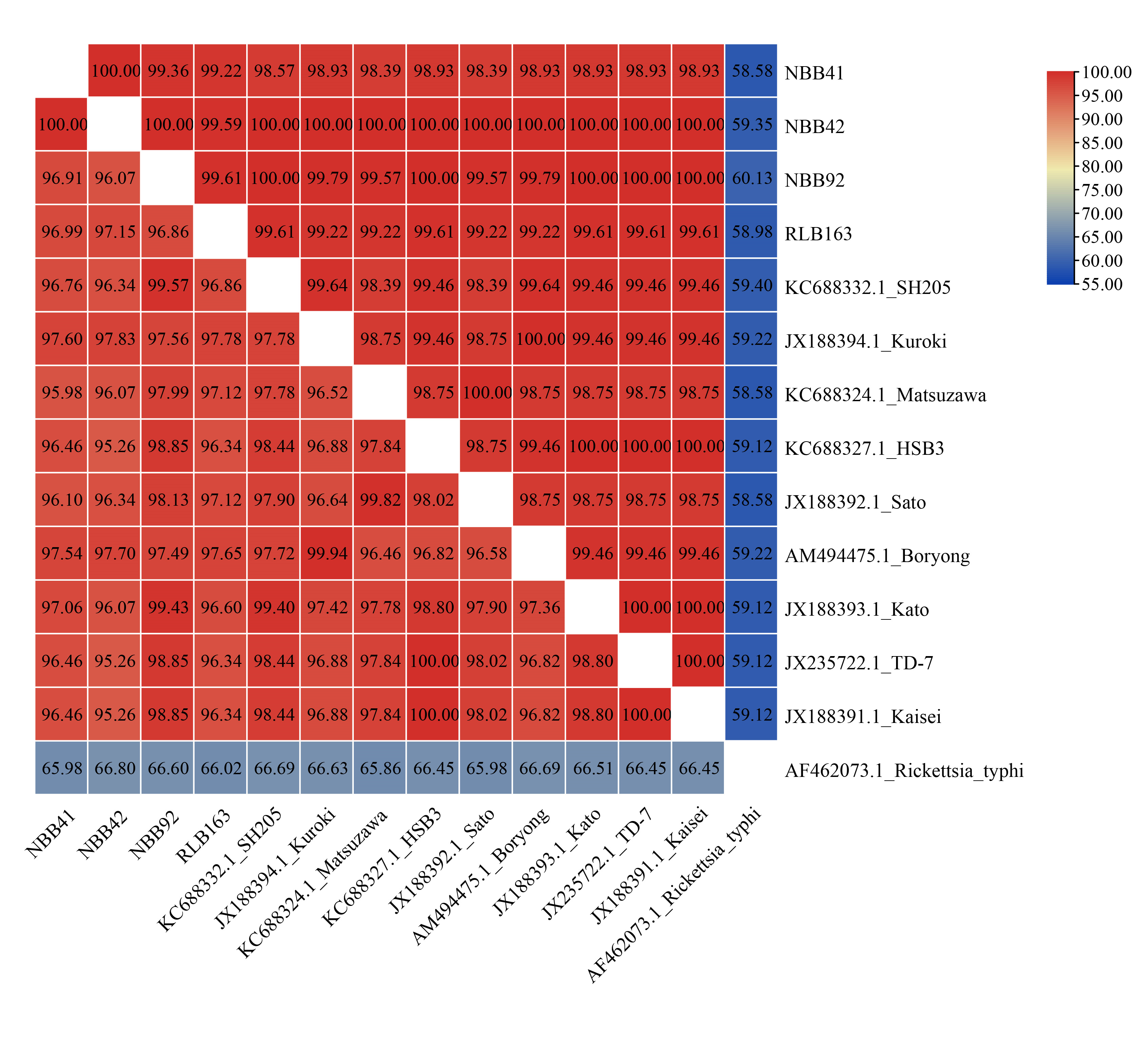

Supplement: S3 Fig — Top right indicates acid sequence identity, while bottom left shows nucleotide sequence identity. (TIF) [file pntd.0013860.s006.tif]

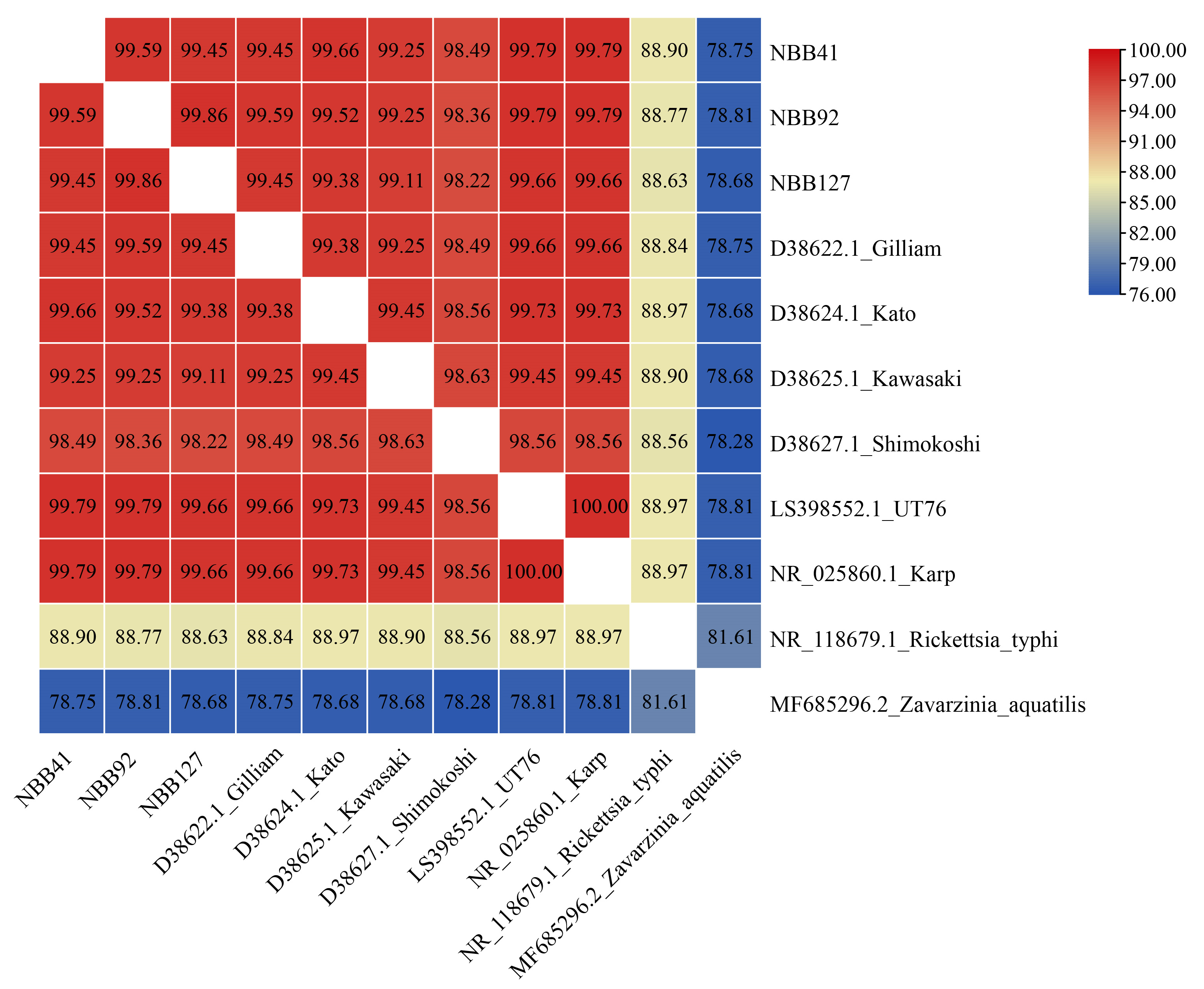

Supplement: S4 Fig — (TIF) [file pntd.0013860.s007.tif]
