# Supplementary material for: Molecular detection of Orientia tsutsugamushi infection in bats from the China-Myanmar border
Source: PLoS Negl Trop Dis. 2026 Jan 12;20(1):e0013860. doi: 10.1371/journal.pntd.0013860 (PMC12818740; doi:10.1371/journal.pntd.0013860)
Supplement: Attachment — Submitted filename: review comments.docx [file pntd.0013860.s008.docx]

The article describes the detection of *Orientia tsutsugamushi* (Ot) in bat tissue samples. Since information on the infection of non-rodent animals are scarce, the presented data is scientifically relevant.

Title

“First discovery” is a tautology. A study from South Korea describes 12% *Orientia*-seropositive bats (see below), so I do not see this as a discovery. Independently, the “firstness” of a description is scientifically not as relevant as the finding itself.

“Bat-borne” is misleading since it indicates transmission, the report describes “bat infection”.

The determination of “origin” is based on a single phylogenetic algorithm, this wording is too pretentious for the title. An example for a more accurate title could be: “Molecular detection of *Orientia tsutsugamushi* infection in bats from the China-Myanmar border”.

Introduction

L50: A more modern concept of orientiae is that different species cause scrub typhus worldwide, TD is only the infection caused by OT.

L54: The reclassification is historical and can be erased.

L62: replace “several” by “severe”

L74: link is not working

L75: Replace or erase “traditionally”, unclear which tradition you refer to

L78: “the disease”: you mean “scrub typhus” since TD is defined to the triangle

L79-80: the sentence can be erased thins these 10 cases are not relevant

L81: erase “additionally”. Distinct Orientia species causing human disease have been detected in southern Chile, the Middle East and countries in Africa. The finding in France requires confirmation.

L90ff: Since you mention mites as reservoir and vector above, I would erase here and focus in more detail on the infection of animals (rodents and others). This is the main topic of the paper and should be elaborated in more detail here and in the discussion (e.g. reviews of Traub 1974, Elliott 2019, and others). Elliott mentions a study describing seropositive bats (*Rhinolophus ferrumequinum*, *Eptisicus serotinus* 12% positive) (10.1186/s13071-019-3751-x). The original paper is: Lee et al. K Korean Soc Microbiol 1996; 31(2). This paper should be mentioned.

L95/L96: please erase “no previous” and “first”, since not correct and not scientifically relevant

Revise and name infection/exposure of other animals, dogs, cats, shrews, check for reports in birds and reptiles… Also cite study from USA, which checked for Orientia antibodies in bats 10.3376/1081-1710(2006)31[386:ssoeff]2.0.co;2

Results

Since samples derived from dead bats caught in fruit trees, the isolation of long OT sequences in tissue samples is an exceptional success, compared to some rodent studies. Do the authors have an explanation? This could be mentioned and discussed in the Discussion.

L210: “no prior reports of Ot infection in bats” 🡪 see comment above on bat seropositivity in South Korea!

L233-35: I do not agree that the data suggest neurotropism. As in rodents, the load was rather low and CNS manifestations are most probably related to immune-activation. See and consider citing 0.1371/journal.pntd.0003064.
