# Supplementary material for: Molecular detection of Orientia tsutsugamushi infection in bats from the China-Myanmar border
Source: PLoS Negl Trop Dis. 2026 Jan 12;20(1):e0013860. doi: 10.1371/journal.pntd.0013860 (PMC12818740; doi:10.1371/journal.pntd.0013860)
Supplement: Attachment — Submitted filename: PNTD-D-25-01569_Response to Reviewers.docx [file pntd.0013860.s010.docx]

Dear Editors,

We sincerely thank you and the three reviewers for your thoughtful and constructive feedback on our manuscript, “*Molecular detection of Orientia tsutsugamushi infection in bats from the China-Myanmar border*” (**PNTD-D-25-01569**). We have carefully considered each of the reviewers’ comments and have made extensive revisions to the manuscript. We are now submitting a revised version for your review, which we believe addresses all the concerns raised and significantly improves the manuscript. We are confident that the revised manuscript is now in appropriate form for publication in *PLOS Neglected Tropical Diseases*.

Below, we provide a point-by-point response to the reviewer’s comments, with our responses highlighted in red.

**Responses to Comments from Reviewer #1:**

1. If indeed this is the first detection of Orientia in bats, it is an interesting observation.

**RESPONSE:** Thank you for your positive comment. We highly appreciate your recognition of the potential value of our study. To clarify the novelty of our research, we have updated the manuscript to specify that this study is the first global report of ***Orientia* DNA detection** in bats. Although previous studies have detected ***Orientia* antibodies** in two bat species in South Korea (1996), this is the first **molecular detection** of the pathogen in bat tissues. We have revised both the Introduction and Discussion sections to address this point. Thank you again for your helpful feedback, which enabled us to refine our manuscript’s presentation and accurately communicate the novelty of our study.

2. The authors present their findings as if bats play a role in the zoonotic cycle of Orientia tsutsugamushi. Bats and all other animal hosts are accidental dead-end hosts of Orientia tsutsugamushi. Orientia are maintained solely by transovarian transmission in mites. The concept that strains of Orientia have evolved in bats is inaccurate.

**RESPONSE:** Thank you for your insightful comment. You are absolutely correct, and we have revised the manuscript accordingly. We now clarify that bats in this study are accidental dead-end hosts of *Orientia tsutsugamushi* (Ot). Existing research has shown that *Leptotrombidium delicense* and *Leptotrombidium fletcheri* (chigger species that can transmit Ot) have been found on bats, leading us to hypothesize that bats may serve as dispersal agents, transporting Ot-positive chiggers to new areas. We have amended the manuscript to reflect this understanding, replacing "due to bites by Ot-carrying chiggers" with "due to accidental bites by Ot-carrying chiggers." These revisions are found in lines 231, 235-241.

3. The author's concept that Orientia is primarily transmitted by chiggers suggests that the other modes of transmission, which include only transplacentalal, transfusion, and organ transplantation, have greater importance than they do.

**RESPONSE:** Thank you for this important clarification. We agree that transmission via blood transfusion and organ transplantation is more relevant to clinical settings and does not apply to wild bat populations. We have revised the manuscript to clarify that the observed infections in bats are likely due to accidental bites from Ot-carrying chiggers. In addition, we have supplemented the introduction to accurately describe the primary modes of transmission of Ot. Please see lines 53-54.

4. The fact that analysis of different genes puts the same strain in different clades indicates that the clades of these Orientia are not sufficiently determined and the analyses are not useful.

**RESPONSE:** Thank you for raising this point. In the revised manuscript, we have removed the explanation regarding the differing classifications of strains into distinct clades and instead added the following clarification: "This discrepancy may indicate that the clades of these *Orientia* are not sufficiently determined." This revision can be found in lines 249-250.

5. The low number of copies of the 47 kDa gene in the organs suggests that some organs were probably only measuring Orientia in the blood in the organ rather than infection of the cells in the organ. The authors do not report the concentration of Orientia in the blood.

**RESPONSE:** We appreciate this observation. Since all bat samples were collected post-mortem, we could not obtain fresh blood samples for quantification of Ot in the blood. We acknowledge that the detected levels may reflect residual Ot in the blood within the organs rather than intracellular infection. However, the quantitative data presented in the study provides reference values for future research on the distribution of Ot in bat tissues. We have also noted in the manuscript that future studies will involve live-captured bats and blood sampling to better quantify Ot loads. Please see lines 255-256.

6. Line 208-210: Small mammals do not play a role in the transmission of scrub typhus. They are merely a source of blood for the larval mites.

**RESPONSE:** Thank you. We have revised this section to reflect your comment. Please see lines 226-227 for the updated text.

7. The molecular clock analysis is unlikely to reflect evolution related to the accidental dead-end infection of bats.

**RESPONSE:** Thank you for your comment. We have clarified the limitations of our molecular clock analysis in the revised manuscript. Given the limited research on Ot in bats, we have reframed this section to focus on the "origins of bat infections," rather than suggesting it reflects the evolution of dead-end infections. The limitations are now explicitly acknowledged in lines 262-265.

**Responses to Comments from Reviewer #2:**

1. There are inconsistencies between the Methods and Results sections; for instance, the reported target sequence length does not match the obtained sequence length that was subsequently analyzed. This discrepancy should be clarified. In addition, the description of the nested PCR procedure is insufficient. Providing detailed methodological information is essential to ensure reproducibility of the study.

**RESPONSE:** Thank you for pointing this out. We have clarified that the target sequence lengths reported in the Methods section correspond to the assembled fragments in the Results section. We have also provided a clearer explanation of the nested PCR procedure in the supplementary tables (S2 and S3). Please see lines 138-139 for the revision.

2. The manuscript does not describe the habitat range of the studied bat species or their potential ecological interactions with chigger mites. Such information is crucial to evaluate the role of bats in long-distance dispersal of O. tsutsugamushi and would significantly strengthen the novelty and conclusions of the study.

**RESPONSE:** Thank you for this valuable suggestion. We have added details about the habitat range of the studied bat species and their potential ecological interactions with chigger mites in the Discussion section. This information is found in lines 235-243.

3. Furthermore, the conclusions drawn from the molecular clock analysis may be premature, as they are based on a limited number of sequences, including some that are not clearly described. A more detailed explanation of the sequence selection and validation would help to strengthen this part of the study.

**RESPONSE:** Thank you for this feedback. We have added more detail on sequence selection and validation in the Methods section (lines 338-342). The molecular clock analysis remains an exploratory approach, and we have clarified this in the manuscript.

**Responses to Comments from Reviewer #3:**

1. The article describes the detection of *Orientia tsutsugamushi* (Ot) in bat tissue samples. Since information on the infection of non-rodent animals are scarce, the presented data is scientifically relevant.

**RESPONSE:** Thank you for your positive recognition of our study's significance. Your support is greatly appreciated, and we have made further revisions to emphasize the novelty and importance of our findings.

2. “First discovery” is a tautology. A study from South Korea describes 12% Orientia-seropositive bats (see below), so I do not see this as a discovery. Independently, the “firstness” of a description is scientifically not as relevant as the finding itself. “Bat-borne” is misleading since it indicates transmission, the report describes “bat infection”. The determination of “origin” is based on a single phylogenetic algorithm, this wording is too pretentious for the title. An example for a more accurate title could be: “Molecular detection of Orientia tsutsugamushi infection in bats from the China-Myanmar border”.

**RESPONSE:** Thank you for your thoughtful suggestion. We have revised the title to: *Molecular detection of Orientia tsutsugamushi infection in bats from the China-Myanmar border*, which we believe more accurately reflects the focus of the study. The term “first” has been removed, and the manuscript has been adjusted accordingly.

3. L50: A more modern concept of orientiae is that different species cause scrub typhus worldwide, TD is only the infection caused by OT.

**RESPONSE:** “also known as scrub typhus” has been removed.

4. L54: The reclassification is historical and can be erased.

**RESPONSE:** We have removed the historical reclassification from the manuscript as suggested.

5. L62: replace “several” by “severe”

**RESPONSE:** The word “several” has been changed to “severe” in line 65.

6. L74: link is not working

**RESPONSE:** We have replaced the link, which should now work correctly. Please see lines 77 and 78.

7. L75: Replace or erase “traditionally”, unclear which tradition you refer to

**RESPONSE:** We have removed the word "traditionally".

8. L78: “the disease”: you mean “scrub typhus” since TD is defined to the triangle

**Response:** We thank the reviewer for pointing this out. We have changed “the disease” to “scrub typhus” in line 81.

9. L79-80: the sentence can be erased thins these 10 cases are not relevant

**RESPONSE:** We have removed the sentence.

10. L81: erase “additionally”. Distinct Orientia species causing human disease have been detected in southern Chile, the Middle East and countries in Africa. The finding in France requires confirmation.

**RESPONSE:** We have removed “additionally”.

11. L90ff: Since you mention mites as reservoir and vector above, I would erase here and focus in more detail on the infection of animals (rodents and others). This is the main topic of the paper and should be elaborated in more detail here and in the discussion (e.g. reviews of Traub 1974, Elliott 2019, and others). Elliott mentions a study describing seropositive bats (*Rhinolophus ferrumequinum*, *Eptisicus serotinus* 12% positive) (10.1186/s13071-019-3751-x). The original paper is: Lee et al. K Korean Soc Microbiol 1996; 31(2). This paper should be mentioned.

**RESPONSE:** As suggested by the reviewer, we have removed “*Ot has predominantly been detected in humans, rodents, and chigger mites*”. Additionally, the infection of animals (rodents and others) has been added in the Introduction section in lines 91-104, and in the Discussion section in lines 219-223. We have mentioned and cited relevant references.

12. L95/L96: please erase “no previous” and “first”, since not correct and not scientifically relevant

**RESPONSE:** We have removed “first”, and have changed “no previous” to “scarce” in line 109.

13. Revise and name infection/exposure of other animals, dogs, cats, shrews, check for reports in birds and reptiles… Also cite study from USA, which checked for Orientia antibodies in bats 10.3376/1081-1710(2006)31[386:ssoeff]2.0.co;2

**RESPONSE:** Thanks. The infection or exposure status in other animals has been supplemented, please see lines 94-97. Reports on the infection of birds and reptiles with this pathogen have been verified, please see lines 97-100. We have now cited a U.S. study detecting *Orientia* antibodies in bats, please see lines 103-104.

14. Since samples derived from dead bats caught in fruit trees, the isolation of long OT sequences in tissue samples is an exceptional success, compared to some rodent studies. Do the authors have an explanation? This could be mentioned and discussed in the Discussion.

**RESPONSE:** We thank the reviewer for this valuable suggestion. Indeed, research on rodents is relatively extensive, but most studies employ universal primers for amplification, yielding relatively short sequences. Previously, the DNA of Ot had not been detected in bats. We designed primers for full-length amplification of four genes—56-kDa TSA, 47-kDa *htrA*, GroEL, and 16S rRNA—using DNA walking amplification. Consequently, we obtained the sequences longer than most previously reported Ot sequences from rodents.

15. L210: “no prior reports of Ot infection in bats” 🡪 see comment above on bat seropositivity in South Korea!

**RESPONSE:** We have changed this sentence in line 227.

16. L233-35: I do not agree that the data suggest neurotropism. As in rodents, the load was rather low and CNS manifestations are most probably related to immune-activation. See and consider citing 0.1371/journal.pntd.0003064.

**RESPONSE:** We agree with the reviewer, thus have removed the statement regarding the neurotropism of Ot. Instead, we have added information, describing that CNS manifestations are most probably related to immune activation in lines 252-255.
