# Supplementary material for: Molecular detection of Orientia tsutsugamushi infection in bats from the China-Myanmar border
Source: PLoS Negl Trop Dis. 2026 Jan 12;20(1):e0013860. doi: 10.1371/journal.pntd.0013860 (PMC12818740; doi:10.1371/journal.pntd.0013860)
Supplement: Attachment — Submitted filename: PNTD-D-25-01569R2_Response to Reviewers.docx [file pntd.0013860.s011.docx]

Dear Editors,

We sincerely thank you and the two reviewers for your thoughtful and constructive feedback on our manuscript, “*Molecular detection of Orientia tsutsugamushi infection in bats from the China-Myanmar border*” (**PNTD-D-25-01569R1**). We have carefully considered each of the reviewers’ comments and have made extensive revisions to the manuscript. We are now submitting a revised version for your review, which we believe addresses all the concerns raised and significantly improves the manuscript. We are confident that the revised manuscript is now in appropriate form for publication in *PLOS Neglected Tropical Diseases*.

Below, we provide a point-by-point response to the reviewer’s comments, with our responses highlighted in red.

**Responses to Comments from Reviewer #1:**

1. The authors have responded appropriately in part to the previous critiques. However, the text is inconsistent in its responses and needs to be harmonized.

**RESPONSE:** Thank you for pointing out the inconsistencies. In line with your suggestions, we have addressed the comments you raised with revisions and responses to eliminate such inconsistencies and ensure all explanations are coherent and consistent. We appreciate your constructive feedback.

2. Lines 19 and 40: It is misleading to state that Orientia tsutsugamushi is mainly or primarily transmitted by feeding chiggers. It is nearly exclusively chigger-transmitted. Deletion of the words "mainly" and "primarily" would be appropriate.

**RESPONSE:** Thank you. We have removed the words "mainly" and "primarily".

3. Lines 32, 47: The term "bat-derived" could be interpreted as Orientia tsutsugamushi having evolved in bats. This is not true. The strains are mite-derived. It would be more appropriate to state "Orientia tsutsugamushi isolated from bats" than "bat-derived".

**RESPONSE:** Thank you. We have replaced the term "bat-derived" with "Ot isolated from bats" in lines 30,43.

4. Lines 34, 46, 208-214: Orientia tsutsugamushi has tropism mainly for endothelial cells and less so for macrophages. These cells infected with Orientia tsutsugamushi are present in all organs. The bacterial loads described are likely also affected by circulating Orientia tsutsugamushi in blood. In valid animal models the lungs are a major target.

**RESPONSE:** Thank you. Corresponding descriptions related to tissue tropism have been removed from the Abstract and Author Summary sections, with additional explanations and revisions made in the Discussion section in lines 254-258.

5. Lines 35-37: The findings reported in this manuscript do not raise new questions regarding the role of bats in transmission. Bats play no role in transmission of Orientia tsutsugamushi.

**RESPONSE:** Thank you. “raising new questions about their role in the transmission ecology of TD” has been deleted.

6. Lines 89-90: It should be noted that Orientia chuto has been detected in southwestern Asia and eastern Africa.

**RESPONSE:** Thank you. “Notably, *Orientia chuto* has been detected in southwestern Asia and eastern Africa.” This sentence has been added to the Introduction section in lines 81-82.

7. Lines 99-101: Rodents and other mammals are not reservoirs of Orientia tsutsugamushi. They are dead-end hosts.

**RESPONSE:** Thank you. We have revised the description referring to rodents and other mammals as reservoirs to dead-end hosts, please see lines 95.

8. Lines 186 and 188: It would be appropriate to remove the designation China. Taiwan is sufficient.

**RESPONSE:** Thank you for your suggestion. However, we respectfully maintain the designation of "China" preceding "Taiwan" in the manuscript. This is because Taiwan is an inalienable part of China's territory. In accordance with the one-China principle, it is both accurate and necessary to clarify the geographical and administrative affiliation by specifying the term "Taiwan, China".

9. Line 237: Chiggers feed on tissue fluid from the dermis, not blood.

**RESPONSE:** Thank you. The sentence beginning with "While small…" has been removed.

**Responses to Comments from Reviewer #3:**

1. L219: “demonstrate the detection” = bad style. The sentence refers to China or worldwide? Not clear how this sentence connects to the previous…

**RESPONSE:** Thank you. The sentence refers to worldwide, with “Worldwide” added in line 221. The previous sentence compares Ot detection in bats with that in rodents. This sentence specifies in which animals Ot has been detected, leading into the next sentence about Ot detection in bats. We added “Additionally” at line 223 to ensure smoother transition between the two statements.

2. L221: “Specifically” is unclear wording here. Explain that you are talking about bats…

**RESPONSE:** Thank you. We have changed “Specifically” to “Additionally” to better connect the preceding sentence with this one in line 223, and added “Ot has been identified in two bat species” to clarify that this sentence is talking about bats. Please see lines 223-224.

3. L225: “can infect”, replace by “infected”

**RESPONSE:** Thank you. We have replaced "can infect" with "infected". Please see line 228.

4. L226: Chiggers do not feed on blood. The entire sentence seems not to make much sense. Better erase the sentence starting with “While small…”.

**RESPONSE:** Thank you. The sentence beginning with "While small…" has been removed.

5. L231: how to conclude that the bites were accidental? You do not know the mite species involved.

**RESPONSE:** Thank you. We removed the term "accidental" from the sentence. What we intend to illustrate in this sentence is that infection with Ot in bats is likely attributable to bites by Ot-carrying chiggers. Given that we did not obtain information on the specific mite species, this is merely a speculation, hence the use of the word "likely".

6. L235: Start sentence with “As other small animals, bats…“

**RESPONSE:** Thank you. The sentence has been revised to start with "As other small animals, bats…"; please refer to lines 235-236.

7. L238: erase “for dispersal”

**RESPONSE:** Thank you. The phrase "for dispersal" has been deleted.

8. L253ff: erase the sentence “the associated…”. There are no CNS manifestations in bats and the findings in dead bats cannot be used to explain human disease in a half sentence.

**RESPONSE:** Thank you. The sentence "the associated…" has been deleted.

9. L39: Please erase “But”, since there no contrast to the sentence before.

**RESPONSE:** Thank you. The conjunction "But" has been deleted.

10. L54: “having also been documented” can be erased to improve style

**RESPONSE:** Thank you. The phrase "having also been documented" has been deleted.

11. L81: the word “expanding” is somehow misleading. Better say “…appears to be wider than previously known, as Orientia species have been detected in the Middle East, Africa, and Chile (20-22)”. Erase “suggesting a broader…”.

**RESPONSE:** Thank you. We have revised according to the suggestions. Please see lines 79-80.

12. L91ff: unclear wording mixing hosts for Ot with host for chiggers. I suggest: L91 replace “natural” by “potential”. And “This” by “The”.

**RESPONSE:** Thank you. We have replaced the word "natural" with "potential", and "This" with "The". Please see line 90.

13. L93ff: you have to clarify “critical role”. For what? Small animals are not the primary reservoir for Ot but maintain chigger populations (the primary Ot reservoir). So they get exposed (serology) and infected (PCR) with Ot. Sorry, this part has to be re-written. The reptiles sentence can be erased.

**RESPONSE:** Thank you. In the revised manuscript, we have clarified the term "critical role" of small mammals. Please see lines 92-95. The sentence regarding reptiles has been removed.
